# Supplementary material for: Elevator-like movements of prestin mediate outer hair cell electromotility
Source: Nat Commun. 2023 Nov 6;14:7145. doi: 10.1038/s41467-023-42489-8 (PMC10628124; doi:10.1038/s41467-023-42489-8)
Supplement: Supplementary file 1 — Supplementary Information [file 41467_2023_42489_MOESM1_ESM.pdf]

Supplementary Information

Elevator-like movements of prestin mediate outer hair cell electromotility

Makoto F. Kuwabara[1,†], Bassam G. Haddad[2,†], Dominik Lenz-Schwab[1,†], Julia Hartmann[1,†], Piersilvio Longo[2], Britt-Marie Huckschlag[1], Anneke Fuß[1], Annalisa Questino[1], Thomas K. Berger[1], Jan-Philipp Machtens[2,3#], and Dominik Oliver[1,4,5#]

1 Department of Neurophysiology, Institute of Physiology and Pathophysiology, Philipps University Marburg, 35037 Marburg, Germany

2 Institute of Biological Information Processing (IBI-1), Molekular- und Zellphysiologie, and JARA-HPC, Forschungszentrum Jülich, Jülich, Germany.

3 Institute of Clinical Pharmacology, RWTH Aachen University, Aachen, Germany.

4 DFG Research Training Group, Membrane Plasticity in Tissue Development and Remodeling, GRK 2213, Philipps University, Germany

5 Center for Mind, Brain and Behavior (CMBB), Universities of Marburg and Giessen, Germany

† these authors contributed equally
# these authors contributed equally

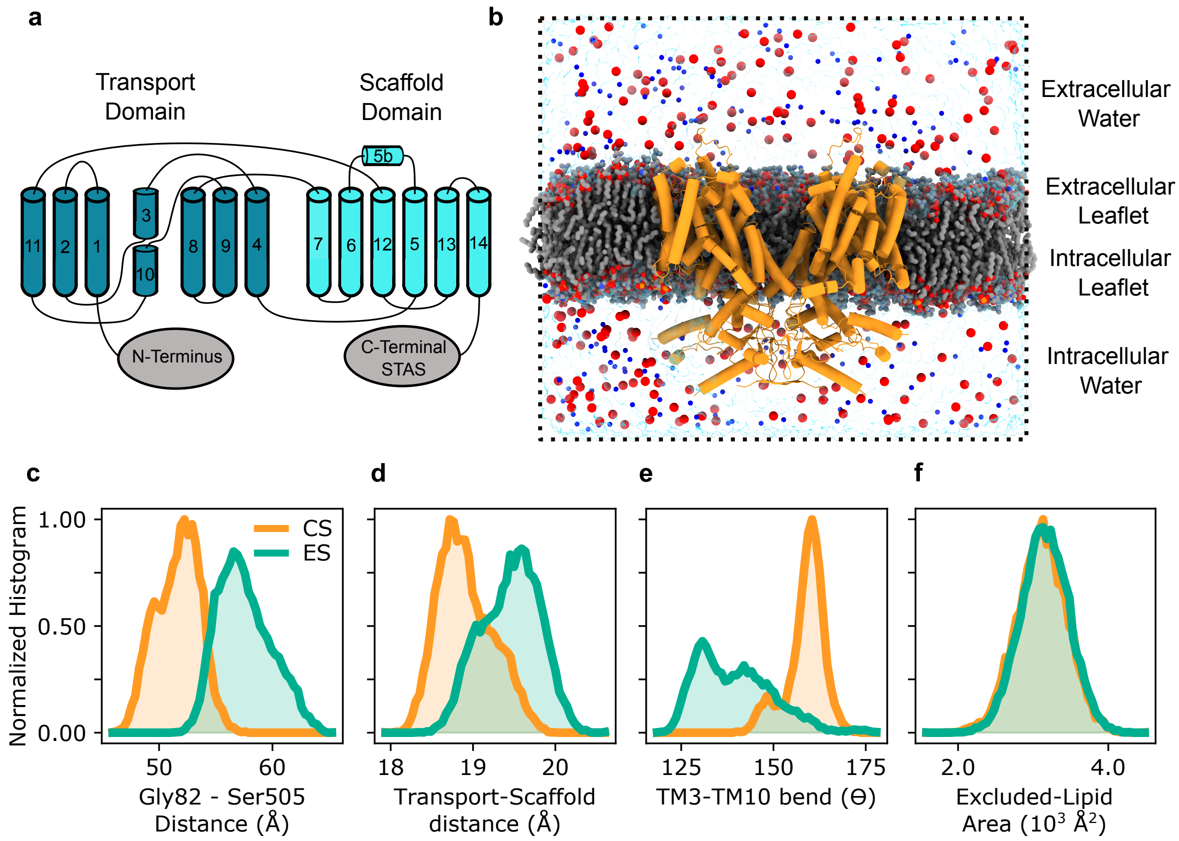

Supplementary Figure 1. Simulation system and structural observables of prestin's TMD.

(a) *Topology of the transmembrane domain (TMD) highlighting the Transport and Scaffold domains within prestin, while exhibiting the 7+7 TM-inverted repeat.*

(b) *MD simulation system: 200 mM NaCl (Na^+: blue, Cl^-: red), fully solvated with TIP3P (light blue), prestin dimer (orange) embedded in a POPC (gray) bilayer. Prestin's orange color denotes its membership to the CS cluster – all simulations initialized in CS.*

(c-f) *Structural observable between the CS and ES clusters.*

(c) *Distribution of Gly82 – Ser505 distance, showing protein expansion in the membrane plane.*

(d) *Separation distance between the centers of geometry (COG) for the Transport and Scaffold respectively.*

(e) *Bending of TM10 relative to TM3 (anion binding pocket).*

(f) *Excluded lipid area as a proxy for membrane expansion within the extracellular leaflet.*

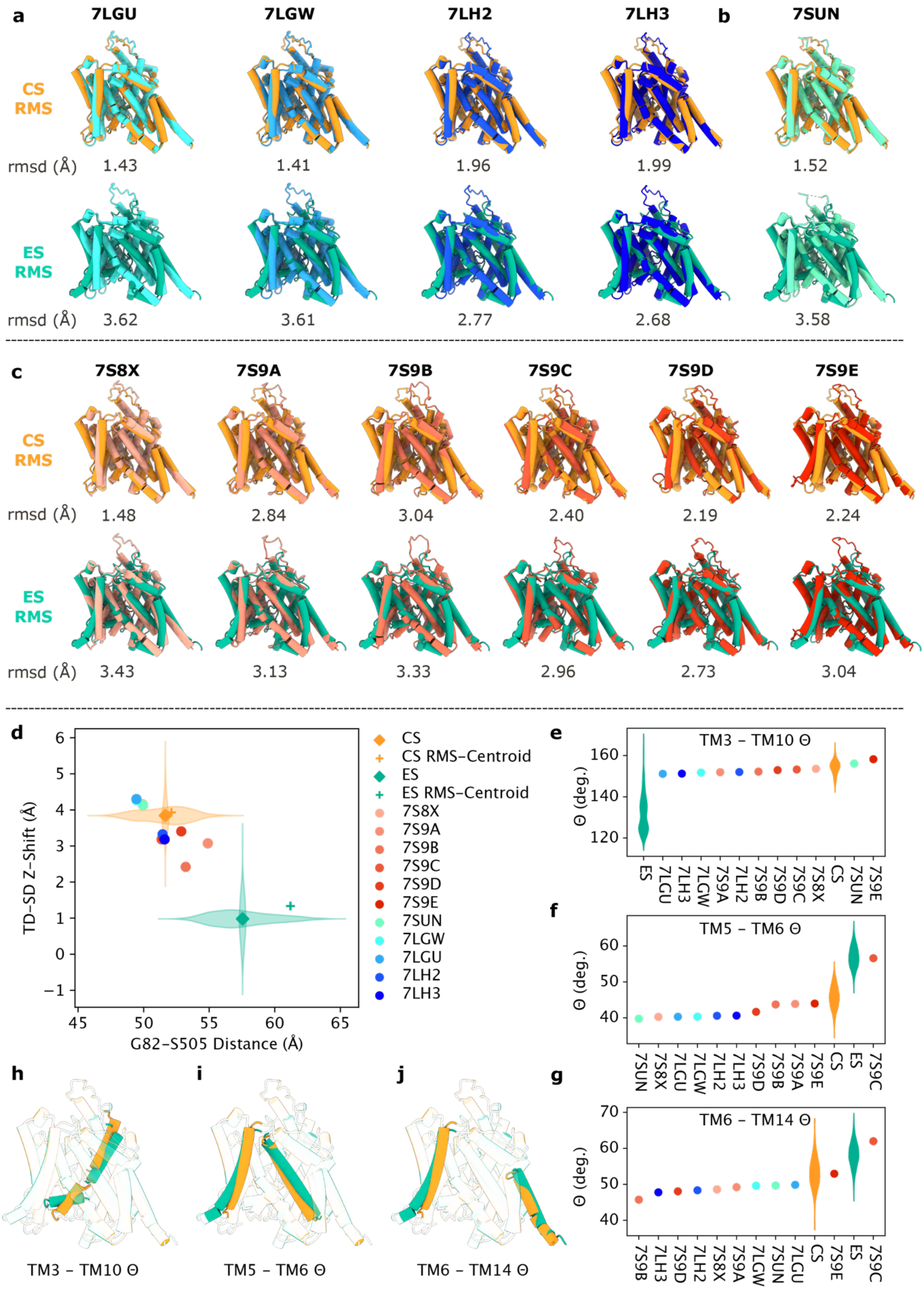
a
7LGU
7LGW
7LH2
7LH3
b
7SUN
CS RMS
rmsd (Å) 1.43 1.41 1.96 1.99 1.52
ES RMS
rmsd (Å) 3.62 3.61 2.77 2.68 3.58
c
7S8X
7S9A
7S9B
7S9C
7S9D
7S9E
CS RMS
rmsd (Å) 1.48 2.84 3.04 2.40 2.19 2.24
ES RMS
rmsd (Å) 3.43 3.13 3.33 2.96 2.73 3.04
d
TD-SD Z-Shift (Å)
G82-S505 Distance (Å)
CS
CS RMS-Centroid
ES
ES RMS-Centroid
7S8X
7S9A
7S9B
7S9C
7S9D
7S9E
7SUN
7LGW
7LGU
7LH2
7LH3
e
TM3 – TM10 Θ
Θ (deg.)
ES 7LGU 7LH3 7LGW 7S9A 7LH2 7S9B 7S9D 7S9C 7S8X CS 7SUN 7S9E
f
TM5 – TM6 Θ
Θ (deg.)
7SUN 7S8X 7LGU 7LGW 7LH2 7LH3 7S9D 7S9B 7S9A 7S9E CS ES 7S9C
g
TM6 – TM14 Θ
Θ (deg.)
7S9B 7LH3 7S9D 7LH2 7S8X 7S9A 7LGW 7SUN 7LGU CS 7S9E ES 7S9C
h
TM3 – TM10 Θ
i
TM5 – TM6 Θ
j
TM6 – TM14 Θ

Supplementary Figure 2. Comparison of Experimental and Simulated Prestin Structures.

(a-c) *Alignment and RMSD of TMD from CS (orange, top) and ES (green, bottom), and prestin structures from [1] (a), [2] (b), and [3] (c). PDBIDs listed in alphabetical order within each respective paper.*

(d) *2D plot of the expansion (G82-S505 Dist.), and TD-SD Z-shift of available WT Prestin structures, and simulated CS (orange) and ES (green) clusters (shown as distributions). For CS and ES, ◆ indicates the geometric centroid of the cluster (i.e. minimized distances in PC-1 and PC-2), where + indicates the RMS-Centroid, which minimizes the RMSD within each respective cluster. The CS-RMS and ES-RMS are used for visualization throughout this manuscript.*

(e-g) *Comparison of TM-angles describing prestin's expansion process. TM-angles were calculated by finding the angle between the principal axes of each respective TM-helix. CS and ES clusters are shown as distributions. TM3 to TM10 (e), TM5 to TM6 (f), TM6 to TM14 (g).*

(h-j) *Cartoon representations of the CS (orange) and ES (green) RMS-Centroids, with the corresponding TMs of interest shown as solid. TM3 and TM10 (h), TM5 and TM6 (i), TM6 and TM14 (j).*

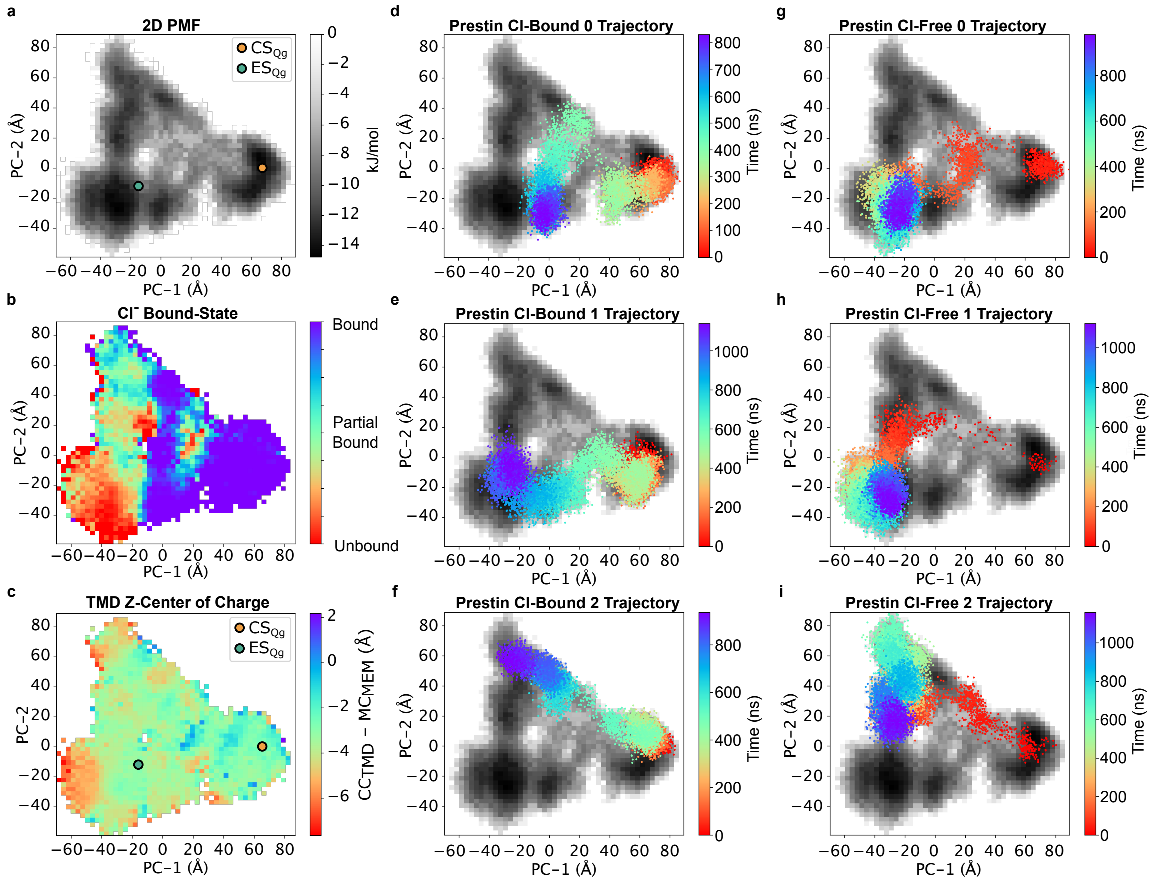

Supplementary Figure 3. Dynamics and characteristics of Prestin Cl-Bound/Free simulations in the Conformational Landscape.

(a) *Conformational landscape of prestin visualized by principal component analysis (PCA) of equilibrium MD simulations. Frequencies are shown as -RTln(δ), where δ is the normalized count in the two-dimensional PCA landscape. This is the same map shown in Figure 1b, without clusters and PDBs plotted. Structures used for constant electric-field calculations projected onto the PCA landscape for the CS (CS_{Qg}) and ES (ES_{Qg}) shown in orange and green, respectively.*

(b) *Chloride-bound state overlaid on PCA conformational landscape. The bound state is defined by the nearest residues to the bound chloride in PDBID: 7LGU (F137, S396, S398). A chloride is considered “Bound” (≡ 2) if it is less than 7 Å from F137 and S396 and S398 Cα atoms, “Partial Bound” (≡ 1) if it is greater than 7 Å from F137 Cα, and less than 7 Å from both S396 and S398 Cα atoms, finally “Unbound” (≡ 0) if the chloride is greater than 7 Å from either S396 or S398 Cα atoms. Non-integer values of the bound state come from averaging within a bin of the 2D conformational landscape.*

(c) *TMD center-of-charge overlaid on PCA conformational landscape. The conformation-dependent charge distribution is evaluated as the difference in the z-component of the charge center (CC) of the TMD (6 e) and the z-component of the mass center (MC) of the membrane; thus, postive values correspond to a movement toward the extracellular solution. Structures used for constant electric-field calculations projected onto the PCA landscape for the CS (CS_{Qg}) and ES (ES_{Qg}) shown in orange and green, respectively.*

(d-f) *Simulation trajectories of Prestin Cl-Bound projected onto the conformational landscape, colored by simulated time. Independent replicates rep-0 (d), rep-1 (e), rep-2 (f).*

(g-i) *Simulation trajectories of Prestin Cl-Free projected onto the conformational landscape, colored by simulated time. Independent replicates rep-0 (g), rep-1 (h), rep-2 (i).*

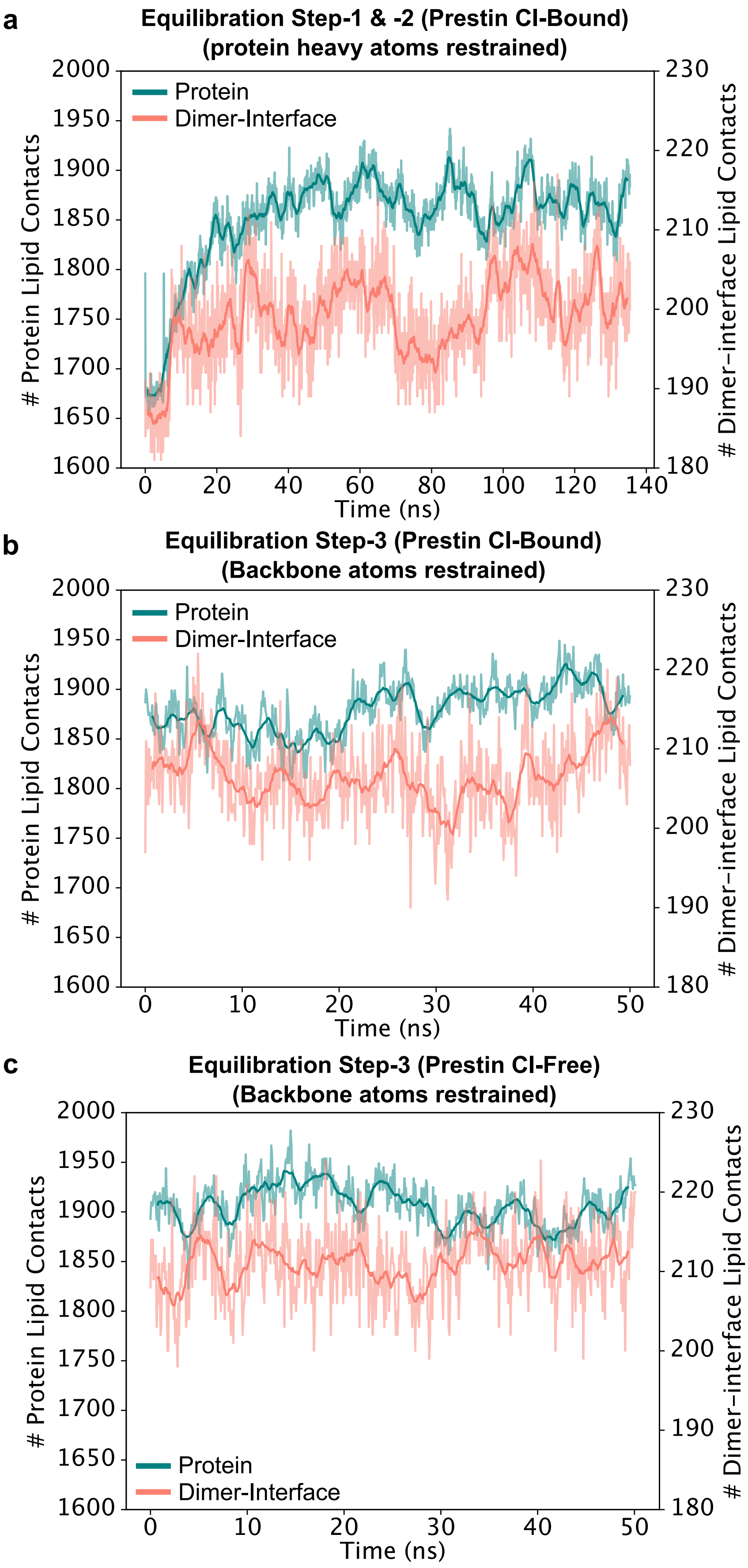
a
Equilibration Step-1 & -2 (Prestin Cl-Bound)
(protein heavy atoms restrained)
Protein
Dimer-Interface
Protein Lipid Contacts
Dimer-interface Lipid Contacts
Time (ns)
b
Equilibration Step-3 (Prestin Cl-Bound)
(Backbone atoms restrained)
Protein
Dimer-Interface
Protein Lipid Contacts
Dimer-interface Lipid Contacts
Time (ns)
c
Equilibration Step-3 (Prestin Cl-Free)
(Backbone atoms restrained)
Protein
Dimer-Interface
Protein Lipid Contacts
Dimer-interface Lipid Contacts
Time (ns)

Supplementary Figure 4. Monitoring equilibration convergence through interactions between lipid headgroup atoms and protein atoms.

A lipid-protein contact is defined as an instance of a protein atom coming within 5 Å of a lipid headgroup atom. Thus, a single protein atom which is within 5 Å of three headgroup atoms (regardless of lipid identity) would constitute 3 contacts.

(a) *Equilibration step-1 and step-2 (see methods) of Prestin Cl-Bound. Step-1, where only lipid tails are free to move, encompasses the first 10 ns and is immediately followed by step-2, where only protein heavy atoms are restrained for the remaining 130 ns.*

(b) *Equilibration step-3 (see methods) of Prestin Cl-Bound. Only protein backbone atoms are restrained.*

(c) *Equilibration step-3 (see methods) of Prestin Cl-Free. The bound chlorides are removed prior to the beginning of this step-3, only protein backbone atoms are restrained.*

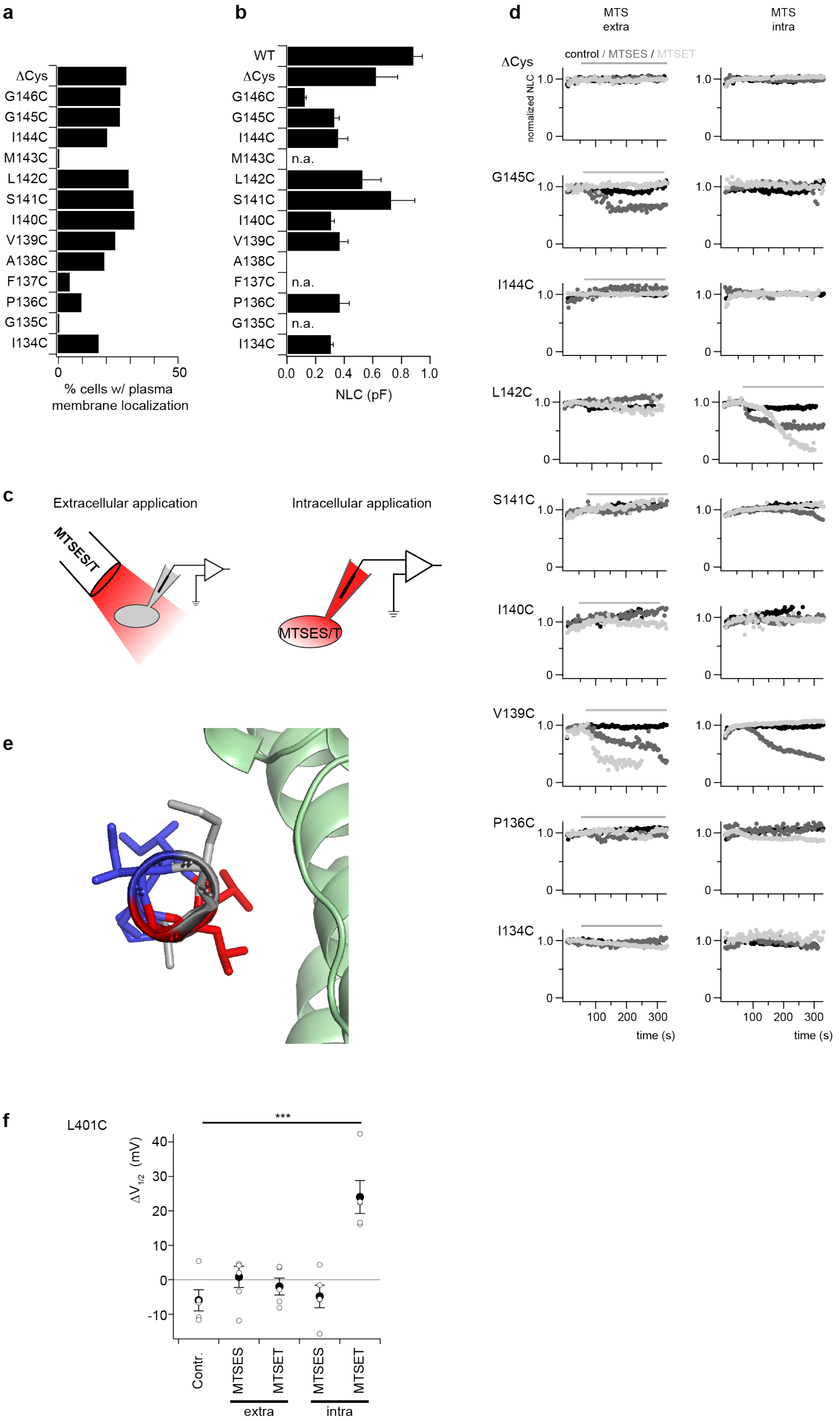
a
b
c
d
e
f
ΔCys
G146C
G145C
I144C
M143C
L142C
S141C
I140C
V139C
A138C
F137C
P136C
G135C
I134C
0
50
% cells w/ plasma membrane localization
WT
n.a.
0.0 0.2 0.4 0.6 0.8 1.0
NLC (pF)
Extracellular application
Intracellular application
MTSES/T
MTS extra
MTS intra
control / MTSES / MTSET
normalized NLC
1.0
100 200 300
time (s)
L401C
***
ΔV1/2 (mV)
40
30
20
10
-10
Contr.
MTSES
MTSET
extra
intra

Supplementary Figure 5: Cysteine scanning mutagenesis in rPres.

(a) *Plasma membrane localization of TM3 cysteine substitution mutants. For each GFP-fused mutant transfected into CHO cells, the total number of fluorescent cells and the percentage of fluorescent CHO cells with visually discernable membrane fluorescence was quantified. The counting was performed on two independent transfections for each mutant and all cells within 5 randomly chosen fields of view on each of four coverslips per transfection was analyzed.*

(b) *Mean (± SEM) NLC of 5 cells, recorded from all mutants that showed robust membrane localization (a).*

(c) *Scheme depicting extra- and intracellular application of MTS compounds during patch-clamp recordings.*

(d) *Representative NLC recordings of MTS-sensitivity from all functional TM3 cysteine substitution mutants. Time course of peak NLC measured every 3s is shown normalized to values after establishment of whole-cell configuration before application of MTSES (dark grey), MTSET (light grey), or control solution (black).*

(e) *Localization of TM3 positions in TM3 responsive to cysteine modification by MTS compounds. Red, MTS sensitivity of NLC amplitude; blue insensitivity to MTS; grey, cysteine substitution mutant is non-functional. Scaffold domain adjacent to TM3 is shown in green, view from the extracellular side.*

(f) *MTSET applied from the intracellular side changes voltage dependence of cysteine substitution mutant L401C. Changes of voltage at peak NLC ($\Delta V_{1/2}$) following application of each reagent are shown as mean ± SEM (black symbols) and individual data points (open circles) from n = 5 cells for each condition.*

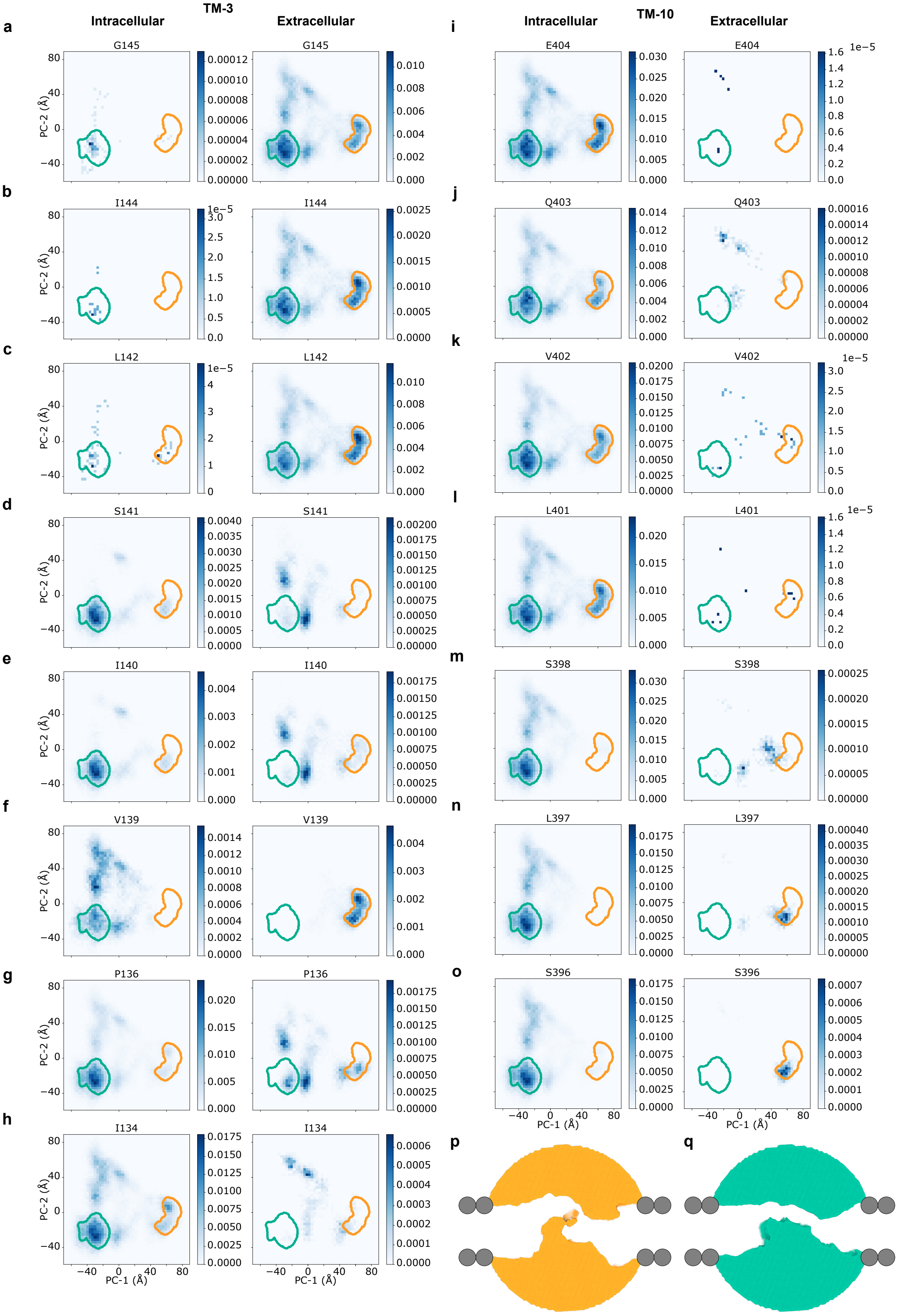
TM-3
Intracellular
Extracellular
TM-10
a
G145
b
I144
c
L142
d
S141
e
I140
f
V139
g
P136
h
I134
i
E404
j
Q403
k
V402
l
L401
m
S398
n
L397
o
S396
p
q
PC-2 (Å)
PC-1 (Å)

Supplementary Figure 6: Per Residue Water Accessibility in prestin

Water-Accessibility is calculated as the average number of water-oxygens within 5 Å of the residue backbone atoms (N – Cα – O) per frame of the simulation (100ps/frame). Outlines for the CS (orange) and ES (green) clusters are overlayed onto water accessibility maps for clarity.

***(a–h)** Per residue water-accessibility for residues in TM3.*

***(i–o)** Per residue water-accessibility for residues in TM10.*

***(p, q)** Average water density map calculated for CS (p) and ES (q). Gray circles represent the location of the membrane.*

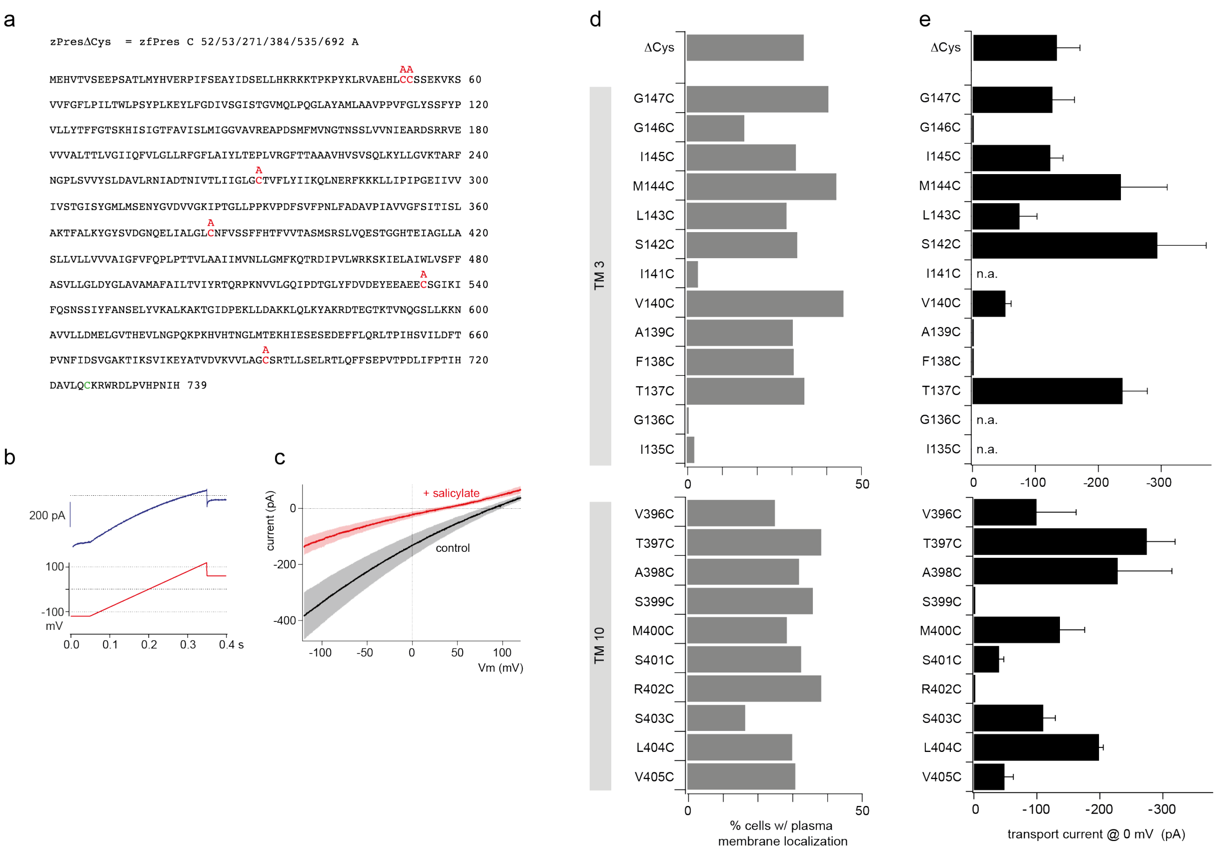

Supplementary Figure 7: Cysteine scanning mutagenesis in zPres.

(a) *Generation of a 'wildtype' variant of zebrafish prestin lacking most endogenous cysteines (*zPres$_{\Delta Cys}$*). Sequence highlights all endogenous cysteines replaced by alanine (red), except for the most C-terminal C726 (green).*

(b) *Recording of oxalate transport current. CHO cell expressing* zPres$_{\Delta Cys}$ *was whole-cell voltage-clamped and subjected to a voltage ramp (-120 to +120 mV, 300 ms; lower panel). Pipette solution contained 106 mM of the substrate anion, oxalate. The resulting current with large inward component and a positive reversal potential indicates robust outward flux of oxalate^{2-} against inward flux of Cl^{-} (upper panel)* [4]*.*

(c) zPres$_{\Delta Cys}$ *generates robust transport currents sensitive to salicylate. Average currents are shown before (black) and during application of salicylate (red; 10 mM, extracellular). N = 6 cells, ± SEM.*

(d) *Plasma membrane localization of cysteine substitution mutants in TM3 and TM10 (upper and lower panels, respectively).* For each GFP-fused mutant transfected into CHO cells, *the total number of fluorescent cells and the percentage of fluorescent CHO cells with visually discernable membrane fluorescence was quantified. The counting was performed on two independent transfections for each mutant and all cells within 5 randomly chosen fields of view on each of four coverslips per transfection was analyzed.*

(e) *Mean (± SEM) transport currents of 5 cells, recorded from all zPres mutants that showed robust membrane localization (a). Currents were obtained from ramp protocols as in (b) at 0 mV to minimize the impact of background and leak current.*

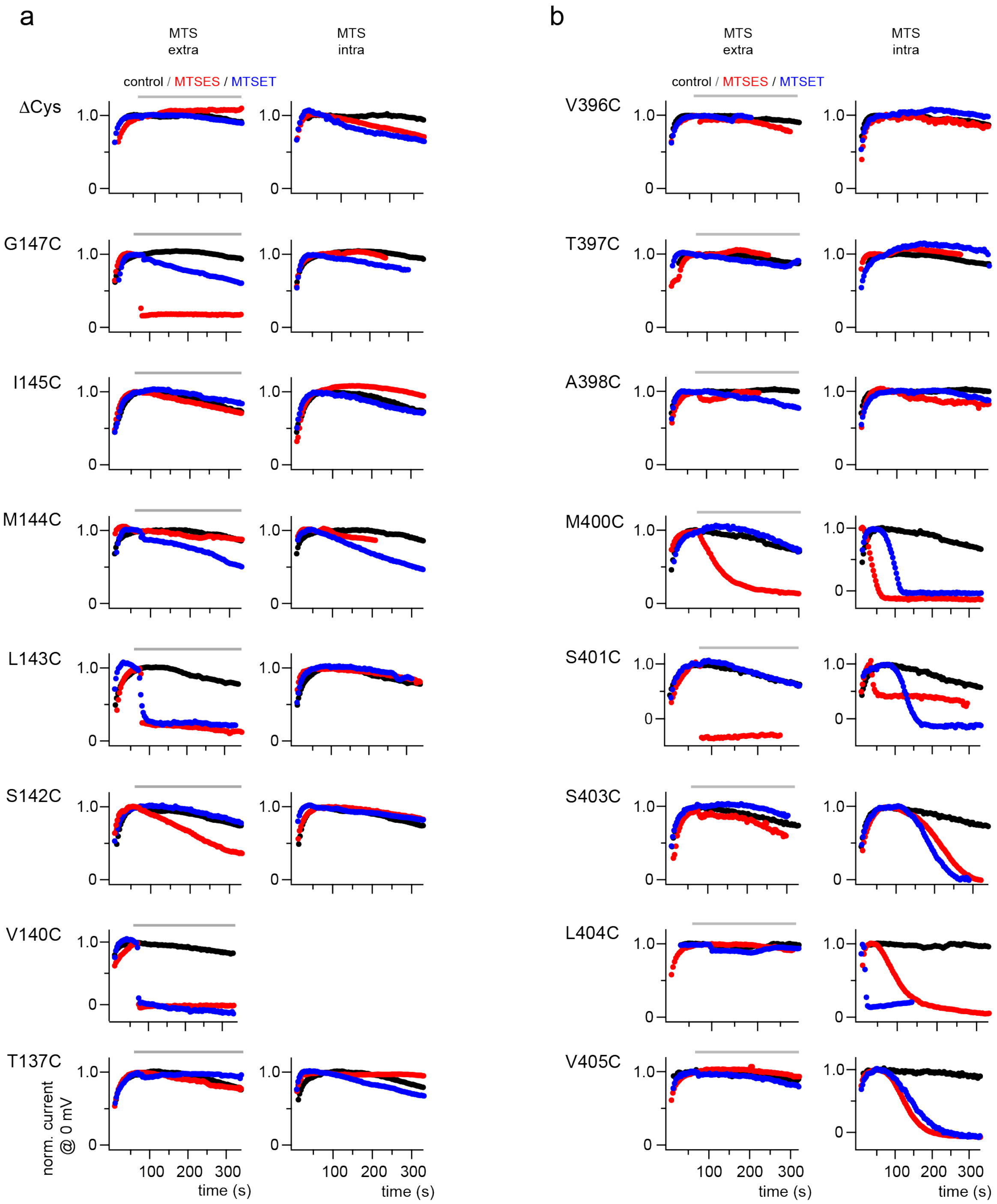

Supplementary Figure 8: Accessibility scanning in zPres.

(a) *Representative recordings of MTS-sensitivity of anion transport activity of all functional cysteine substitution mutants made in TM3 of zPres. Transport current at 0 mV measured by a voltage ramp protocol (see Supplementary Fig. 7b) every 3s is shown normalized to values after establishment of whole-cell configuration before application of MTSES (red), MTSET (blue), or control solution (black). Effects of intracellular MTS compounds on V140C could not be evaluated, because reagents leaking from the pipette tip during approach to the cell extracellularly modified and fully inhibited this mutant before whole-cell recording was initiated.*

(b) *Representative recordings of MTS-sensitivity of anion transport activity of all functional cysteine substitution mutants made in TM10 of zPres.*

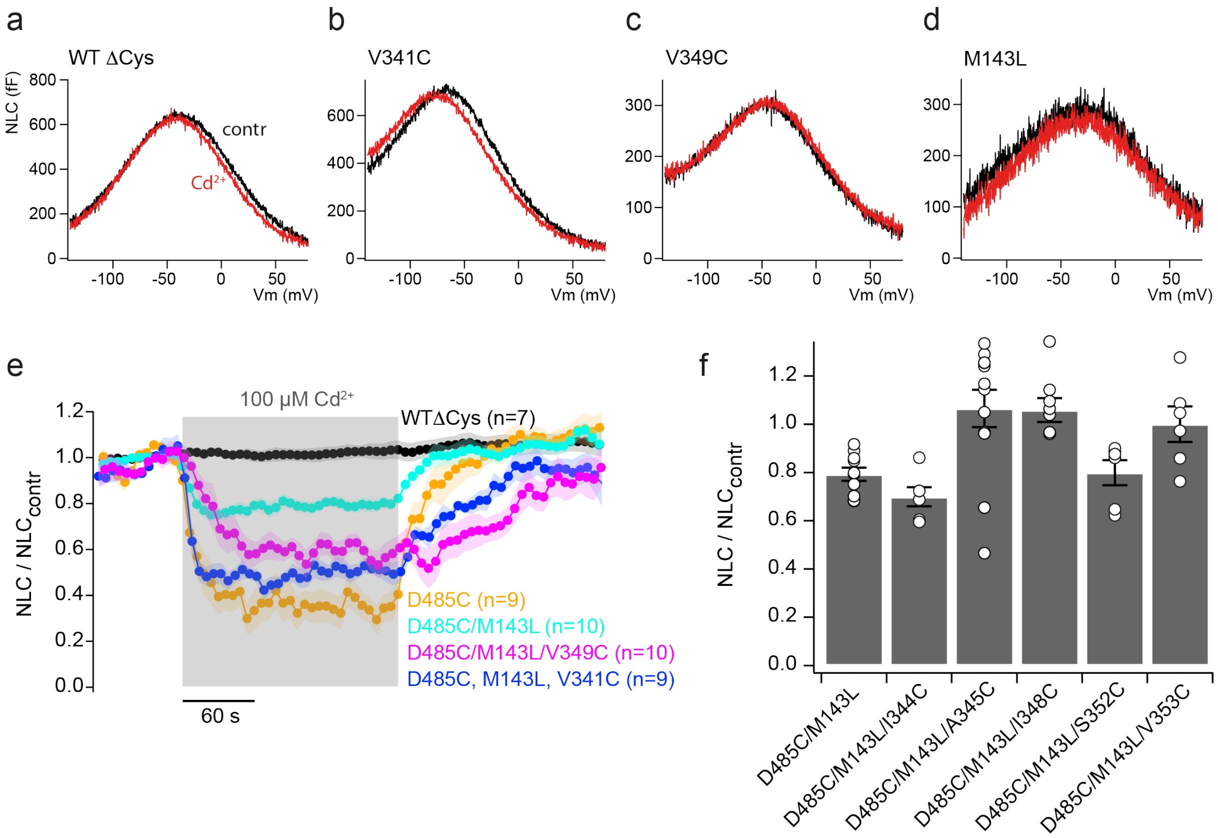

Supplementary Figure 9: Specificity of Cd^{2+} effects in cysteine cross-linking between residue pairs between transport and scaffold domains.

(a-d) *Absence of Cd^{2+} effects on the function of prestin lacking endogenous cysteines (a), single cysteine substitution mutants (b, c), and substitution of endogenous methionine M143 by leucine (d).*

(e) *Time course of inhibition of prestin function by Cd^{2+} in the single, double and triple mutants indicated. Peak NLC is shown normalized to signal amplitude before application of Cd^{2+}.*

(f) *Application of Cd^{2+} did not change the activity of cysteine-pair mutants beyond the minor effect seen with prestin D485C/M143L. Bars show NLC in the presence of Cd^{2} normalized to NLC recorded before the application of Cd^{2} (mean ± SEM). Individual datapoints from independent whole-cell recordings are shown as symbols. Differences to double mutant D485C/M143L were not significant for any of the mutant constructs shown ($p \geq 0.448$; Dunnett's multiple comparison test).*

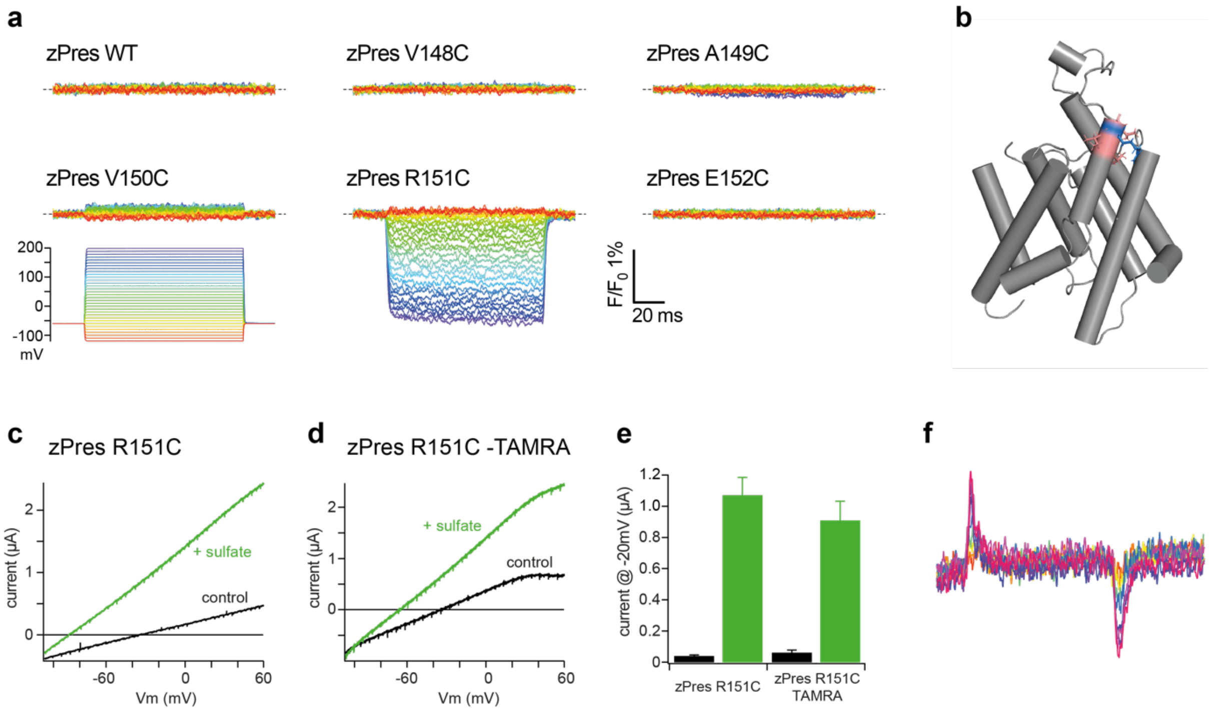

Supplementary Figure 10: Identification of a site reporting voltage-dependent movements of the transport domain by VCF.

(a) *Representative VCF recordings from cysteine substitution mutants of zPres, made in the extracellular end of TM3. zPres mutants were expressed in Xenopus oocytes, covalently labeled with MTS-TAMRA and subjected to the voltage clamp protocol shown in the lower left panel. Fluorescence was excited at 545 nm and detected at 605 nm. Fluorescence intensities were bleaching-corrected and are shown normalized to baseline fluorescence for each individual trace.*

(b) *Location of the cysteine mutations tested for voltage-sensitive TAMRA fluorescence (a). View onto the transport domain from the transport domain–scaffold domain interface (scaffold domain not shown). Positions with minimal or no VCF response are shown in red; responsive position (R151) is highlighted in blue.*

(c, d) *Retained transport function of R151C mutant before (c) and after (d) labeling with MTS-TAMRA. zPres mutant was expressed in Xenopus oocytes and prepared as described for VCF experiments (d). Two-electrode voltage clamp recordings were made with applied voltage ramps (-120 to +60 mV) either without (control) or with 10 mM SO_4^{2-} added to the extracellular solution (see Methods). Robust currents elicited by SO_4^{2-} indicate transport activity.*

(e) *Mean current amplitudes of current at -20 mV (where endogenous background currents are minimal) for each condition, recorded from 10 and 8 oocytes as in (c,d), respectively.*

(f) *Representative capacitive currents meditated by zPres151C-TAMRA recorded from a CHO cell, recorded in response to steps to voltages between -30 and +150 mV. Leak and linear capacitive currents were canceled by using a P/-8 protocol.*

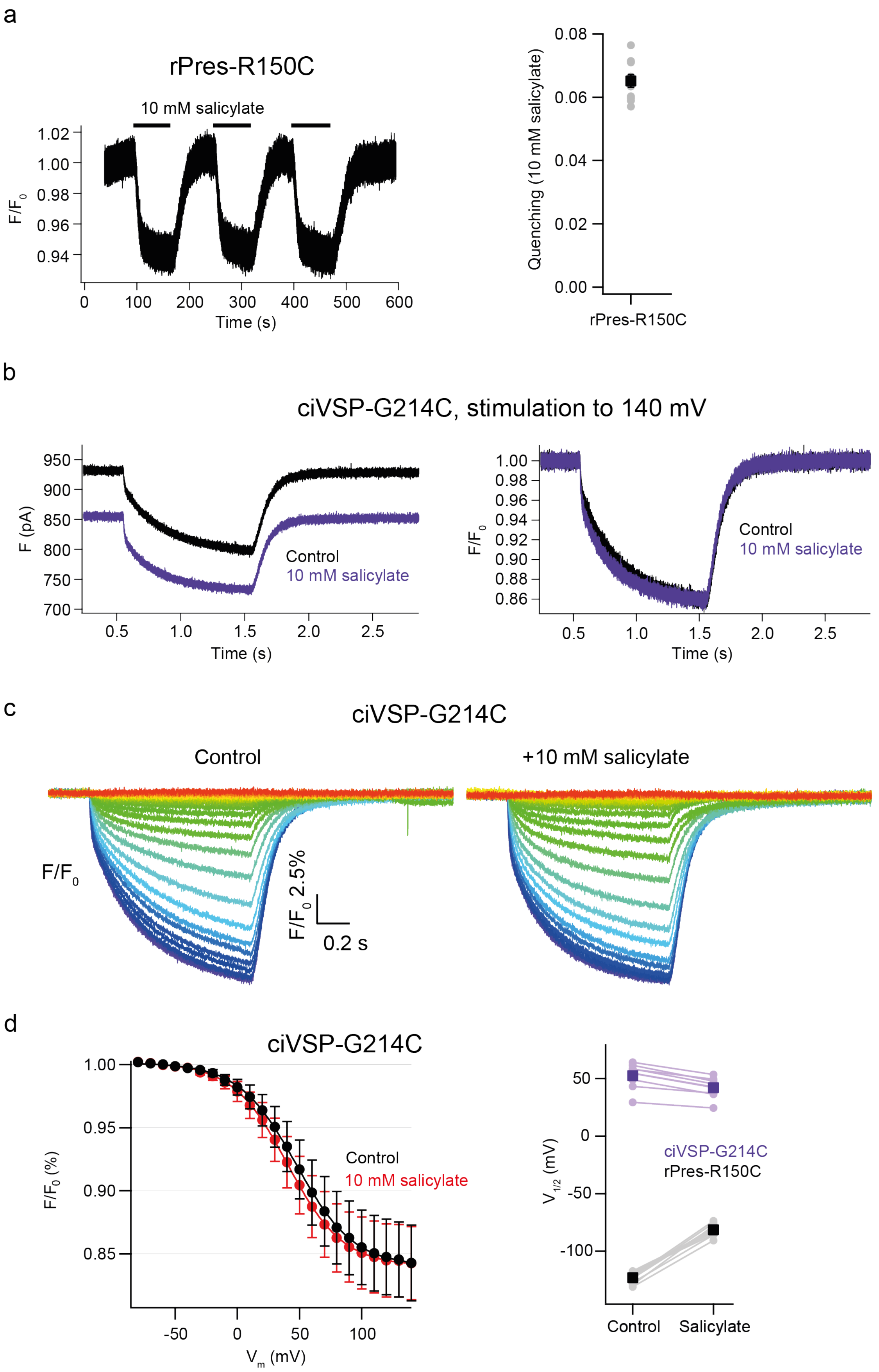
a
rPres-R150C
10 mM salicylate
F/F0
Time (s)
Quenching (10 mM salicylate)
rPres-R150C
b
ciVSP-G214C, stimulation to 140 mV
F (pA)
Control
10 mM salicylate
Time (s)
F/F0
Control
10 mM salicylate
Time (s)
c
ciVSP-G214C
Control
+10 mM salicylate
F/F0
F/F0 2.5%
0.2 s
d
ciVSP-G214C
F/F0 (%)
Control
10 mM salicylate
Vm (mV)
V1/2 (mV)
ciVSP-G214C
rPres-R150C
Control
Salicylate

Supplementary Figure 11: Quenching, and shift of the FV of rPres-R150C, but not of the FV of ciVSP-G214C, to less negative potentials by salicylate.

(a) *Representative VCF recording showing the normalized fluorescence intensity (F/F_0) of TAMRA-labelled rPres-150C in the absence and presence of 10 mM salicylate in the extracellular bath solution at a holding voltage of -30 mV. Fluorescence quenching by 10 mM salicylate, calculated by dividing the change in fluorescence intensity by application of salicylate by fluorescence intensity in the absence of salicylate. Mean ± SEM = 0.065 ± 0.002, n = 10. Grey symbols are individual data points.*

(b) *Left, Representative VCF recording showing the fluorescence intensity (F) of TAMRA-labelled voltage sensitive phosphatase ciVSP-G214C [5] in response to a 1 s voltage step from a holding voltage of -60 mV to +140 mV in the presence and absence of 10 mM salicylate in the extracellular bath solution. The ciVSP mutant also contained the C363S mutation to render the phosphatase domain catalytically inactive [5]. Right, same recording as in the left panel but normalized (F/F_0). The data indicate that salicylate has only a minor impact on the normalized amplitude of the voltage-induced fluorescence changes recorded from ciVSP-G214C. This is in contrast to rPres-R150C, where a specific reduction in F/F_0 is seen in response to the wash-in of 10 mM salicylate at negative membrane potentials (see Fig. 6g,h).*

(c) *Representative VCF recording showing the normalized fluorescence intensity (F/F_0) of TAMRA-labelled ciVSP-G214C in response to a family 1-s voltage steps from -80 mV (red) to up to +140 mV (purple) in 10 mV increments, starting from a holding voltage of -60 mV, and in the absence (left, control) and presence (right) of 10 mM salicylate in the extracellular bath solution. Traces boxcar-filtered (filter width, 1.1 ms).*

(d) *Left, FV curve of ciVSP in the presence (red) or absence (black) of 10 mM salicylate in the extracellular bath solution. Curves were fit with a single Boltzmann with $V_{1/2}$ = 52.6 ± 3.6 mV and α = 21.7 ± 0.4 mV (control) and $V_{1/2}$ = 42.2 ± 2.9 mV and α = 20.7 ± 0.7 mV (10 mM salicylate) (n = 9). Right, comparison of the $V_{1/2}$ values of the FV of the ciVSP-G214C (purple) and rPres-R150C (black) data in the absence and presence of 10 mM salicylate. rPres-R150C data shown here are the same as in Fig. 6i. Note that the substantial shift of $V_{1/2}$ to positive potential is specific to the FVs of rPres-R150C, suggesting that salicylate exerts a specific effect.*

Supplementary Table 1. MD Simulation System Information

	Prestin Chloride-Bound (PCl)			Prestin Chloride-Free (PClF)		
Replicate	0	1	2	0	1	2
Production (ns)	829	1139	936	980	1122	1158
# Atoms		135947			135943	
# Solvent (water)		112642			112642	
# Na^+ (~200 mM)		480			478	
# Cl^- (~200 mM)		502			500	
# Lipids (POPC)		857			857	

Supplementary References:

1 Ge, J. *et al.* Molecular mechanism of prestin electromotive signal amplification. *Cell* **184**, 4669-4679 e4613, doi:10.1016/j.cell.2021.07.034 (2021).

2 Butan, C. *et al.* Single particle cryo-EM structure of the outer hair cell motor protein prestin. *Nature communications* **13**, 290, doi:10.1038/s41467-021-27915-z (2022).

3 Bavi, N. *et al.* The conformational cycle of prestin underlies outer-hair cell electromotility. *Nature* **600**, 553-558, doi:10.1038/s41586-021-04152-4 (2021).

4 Schaechinger, T. J. & Oliver, D. Nonmammalian orthologs of prestin (SLC26A5) are electrogenic divalent/chloride anion exchangers. *Proc Natl Acad Sci U S A* **104**, 7693-7698 (2007).

5 Kohout, S. C., Ulbrich, M. H., Bell, S. C. & Isacoff, E. Y. Subunit organization and functional transitions in Ci-VSP. *Nat. Struct. Mol. Biol.* **15**, 106-108, doi:10.1038/nsmb1320 (2008).
