# Supplementary material for: Elevator-like movements of prestin mediate outer hair cell electromotility
Source: Nat Commun. 2023 Nov 6;14:7145. doi: 10.1038/s41467-023-42489-8 (PMC10628124; doi:10.1038/s41467-023-42489-8)
Supplement: Supplementary file 3 — Reporting Summary [file 41467_2023_42489_MOESM3_ESM.pdf]

nature portfolio

Corresponding author(s): Dominik Oliver, Jan-Philipp Machtens

Last updated by author(s): Oct 10, 2023

Reporting Summary

Nature Portfolio wishes to improve the reproducibility of the work that we publish. This form provides structure for consistency and transparency in reporting. For further information on Nature Portfolio policies, see our Editorial Policies and the Editorial Policy Checklist.

Statistics

For all statistical analyses, confirm that the following items are present in the figure legend, table legend, main text, or Methods section.

n/a	Confirmed	
☐	☒	The exact sample size (*n*) for each experimental group/condition, given as a discrete number and unit of measurement
☐	☒	A statement on whether measurements were taken from distinct samples or whether the same sample was measured repeatedly
☐	☒	The statistical test(s) used AND whether they are one- or two-sided<br>*Only common tests should be described solely by name; describe more complex techniques in the Methods section.*
☒	☐	A description of all covariates tested
☐	☒	A description of any assumptions or corrections, such as tests of normality and adjustment for multiple comparisons
☐	☒	A full description of the statistical parameters including central tendency (e.g. means) or other basic estimates (e.g. regression coefficient) AND variation (e.g. standard deviation) or associated estimates of uncertainty (e.g. confidence intervals)
☐	☒	For null hypothesis testing, the test statistic (e.g. *F*, *t*, *r*) with confidence intervals, effect sizes, degrees of freedom and *P* value noted<br>*Give P values as exact values whenever suitable.*
☒	☐	For Bayesian analysis, information on the choice of priors and Markov chain Monte Carlo settings
☒	☐	For hierarchical and complex designs, identification of the appropriate level for tests and full reporting of outcomes
☒	☐	Estimates of effect sizes (e.g. Cohen's *d*, Pearson's *r*), indicating how they were calculated

Our web collection on statistics for biologists contains articles on many of the points above.

Software and code

Policy information about availability of computer code

Data collection	Gromacs, version 2021 – molecular simulation package (publicly available at https://manual.gromacs.org)<br>Patch Clamp: HEKA Patchmaster (v2x32);<br>Voltage Clamp Fluorometry: WinWCP
Data analysis	Standard python scripts were used to analyze and visualise MD simulations (e.g. water accessibility, structural observables, etc.).<br>IGOR Pro 8 was used to analyze patch-clamp and voltage-clamp fluorometry data, including simple custom-written routines<br>All code used will be made available by the corresponding authors upon request.

For manuscripts utilizing custom algorithms or software that are central to the research but not yet described in published literature, software must be made available to editors and reviewers. We strongly encourage code deposition in a community repository (e.g. GitHub). See the Nature Portfolio guidelines for submitting code & software for further information.

Data

Policy information about availability of data

All manuscripts must include a data availability statement. This statement should provide the following information, where applicable:

- Accession codes, unique identifiers, or web links for publicly available datasets
- A description of any restrictions on data availability
- For clinical datasets or third party data, please ensure that the statement adheres to our policy

Source data for Figures 1b-d, 2a-b, 3b, 4f, and 6j-k are provided with this paper. Initial and final configurations for each simulation replicate are deposited at https://jugit.fz-juelich.de/computational-neurophysiology/prestin_dynamics. All other source data are available from the corresponding authors upon request.
Structural data used: PDB: 7LGU [http://doi.org/10.2210/pdb7LGU/pdb]; PDB: 7S9B [http://doi.org/10.2210/pdb7S9B/pdb]

Research involving human participants, their data, or biological material

Policy information about studies with human participants or human data. See also policy information about sex, gender (identity/presentation), and sexual orientation and race, ethnicity and racism.

Reporting on sex and gender	n/a
Reporting on race, ethnicity, or other socially relevant groupings	n/a
Population characteristics	n/a
Recruitment	n/a
Ethics oversight	n/a

Note that full information on the approval of the study protocol must also be provided in the manuscript.

Field-specific reporting

Please select the one below that is the best fit for your research. If you are not sure, read the appropriate sections before making your selection.

☒ Life sciences ☐ Behavioural & social sciences ☐ Ecological, evolutionary & environmental sciences

For a reference copy of the document with all sections, see nature.com/documents/nr-reporting-summary-flat.pdf

Life sciences study design

All studies must disclose on these points even when the disclosure is negative.

Sample size	Sample size: Regarding MD simulations, at least three independent replicates for each initial condition ensured convergence of all analysed and reported metrics.<br>For electrophysiology, no predetermined sample sizes were calculated. Given previous experience with similar experiments (e.g. DOI: 10.1038/ncomms4622; DOI: 10.1038/emboj.2011.202) and the biophysically reductionistic system studied, we consider the minimum sample size of 5 independent experiments per molecular construct and experimental condition as sufficient. For VCF experiments, the reported sample sizes yielded low variance, indicating sufficient sample size.
Data exclusions	Patch-Clamp electrophysiology: data from individual cells were excluded when Rm < 300 MOhm or Rs > 10 MOhm<br>Regarding MD-simulations, the "cluster based analyses" relied on the DBSCAN clustering algorithm to determine the simulation frames relevant to macro-states.
Replication	Electrophysiology: Recordings were obtained from 5-10 separate cells/oocytes (as detailed in the manuscript) from at least 3 independently transfected/injected batches of cells for each prestin mutant and experimental condition. These replications were pooled for subsequent statistical analysis.<br>MD simulations: Each simulation system, after initial equilibration, were used to generate three independent simulation-replicates. These replicate simulations were combined for subsequent clustering.
Randomization	Electrophysiology: CHO cells were selected for recordings by showing plasma membrane fluorescence (indicating strong expression of GFP-tagged protein and proper protein processing/targeting). Patch-clamp experiment are single-cell experiments, where cells meeting these criteria where chosen randomly. Oocytes were chosen randomly from batches of injected oocytes.<br>MD simulations: Structural analyses of simulations are computed using clusters of pooled data, with cluster assignments determined independently of initial conditions.
Blinding	Electrophysiology/VCF experiments were performed non-blinded (usually the same person performed transfection and experiment) because

Blinding	we expected no performance bias. Specifically, the analysis of quantitative data through analysis routines is robust against influence of experimenter.<br>Blinding is not inherently relevant for molecular/numerical simulations, as computers cannot generate subjective judgments. To this end, other measures have been taken to ensure objectivity in the analysis of the molecular simulations.

Reporting for specific materials, systems and methods

We require information from authors about some types of materials, experimental systems and methods used in many studies. Here, indicate whether each material, system or method listed is relevant to your study. If you are not sure if a list item applies to your research, read the appropriate section before selecting a response.

Materials & experimental systems

n/a	Involved in the study	
☒	☐	Antibodies
☐	☒	Eukaryotic cell lines
☒	☐	Palaeontology and archaeology
☐	☒	Animals and other organisms
☒	☐	Clinical data
☒	☐	Dual use research of concern
☐	☐	Plants

Methods

n/a	Involved in the study	
☒	☐	ChIP-seq
☒	☐	Flow cytometry
☒	☐	MRI-based neuroimaging

Eukaryotic cell lines

Policy information about cell lines and Sex and Gender in Research

Cell line source(s)	CHO cells: ATCC
Authentication	Cell line was not authenticated after receipt from distributor
Mycoplasma contamination	Cell line was tested neagtive for mycoplasma by PCR
Commonly misidentified lines (See ICLAC register)	no commonly misidentified cell lines used

Animals and other research organisms

Policy information about studies involving animals; ARRIVE guidelines recommended for reporting animal research, and Sex and Gender in Research

Laboratory animals	Xenopus laevis
Wild animals	n/a
Reporting on sex	Experiments were done on isolated oocytes. Thus all material was obtained from female frogs.
Field-collected samples	n/a
Ethics oversight	State of Hesse, Regierungspräsidium Gießen, Approval A16/2019

Note that full information on the approval of the study protocol must also be provided in the manuscript.
